# A Lower pH Value Benefits Regeneration of *Trichosanthes kirilowii* by Somatic Embryogenesis, Involving Rhizoid Tubers (RTBs), a Novel Structure

**DOI:** 10.1038/srep08823

**Published:** 2015-03-06

**Authors:** Ke-dong Xu, Yun-xia Chang, Ju Zhang, Pei-long Wang, Jian-xin Wu, Yan-yan Li, Xiao-wen Wang, Wei Wang, Kun Liu, Yi Zhang, De-shui Yu, Li-bing Liao, Yi Li, Shu-ya Ma, Guang-xuan Tan, Cheng-wei Li

**Affiliations:** 1 Key Laboratory of Plant Genetics and Molecular Breeding, Zhoukou Normal University, East Wenchang Street, Zhoukou, 466001, People's Republic of China; 2 College of Life Science and Agronomy, Zhoukou Normal University, East Wenchang Street, Zhoukou, 466001, People's Republic of China

## Abstract

A new approach was established for the regeneration of *Trichosanthes kirilowii* from root, stem, and leaf explants by somatic embryogenesis (SE), involving a previously unreported SE structure, rhizoid tubers (RTBs). During SE, special rhizoids were first induced from root, stem, and leaf explants with average rhizoid numbers of 62.33, 40.17, and 11.53 per explant, respectively, on Murashige and Skoog (MS) medium (pH 4.0) supplemented with 1.0 mg/L 1-naphthaleneacetic acid (NAA) under dark conditions. Further, one RTB was formed from each of the rhizoids on MS medium (pH 4.0) supplemented with 20 mg/L thidiazuron (TDZ) under light conditions. In the suitable range (pH 4.0–9.0), a lower pH value increased the induction of rhizoids and RTBs. Approximately 37.77, 33.47, and 31.07% of *in vivo* RTBs from root, stem, and leaf explants, respectively, spontaneously developed into multiple plantlets on the same MS medium (supplemented with 20 mg/L TDZ) for induction of RTBs, whereas >95.00% of *in vitro* RTBs from each kind of explant developed into multiple plantlets on MS medium supplemented with 5.0 mg/L 6-benzylaminopurine (BAP). Morphological and histological analyses revealed that RTB is a novel type of SE structure that develops from the cortex cells of rhizoids.

T*richosanthes kirilowii* Maximowicz is a perennial unisexual herb. It is grown as an important traditional medicine and economic plant in China. The dried products of male *T*. *kirilowii* roots, a kind of famous traditional Chinese medicine known as “radix trichosanthis”, have been extensively used in the treatment of ectopic pregnancy^1^ and bloat-resistant lesions^2^. Trichosanthin (TCS), the active ingredient of radix trichosanthis, is a type I single chain ribosome-inactivating protein (SCRIP)^3^,^4^ that plays a specific role in inhibiting protein synthesis^5^,^6^.

TCS has widely been used to cure diabetes, rigorous coughing, breast abscesses^7^, hypertension, hyperlipidemia, blood plasmic viscosity^8^, migraines^9^, exfetation, vesicular moles, and ectopic gestation^1^,^10^. TCS has effects on treatments for different tumours and cancers such as malignant tumours^11^,^12^, epithelial cancer^13^, prostatic cancer^14^, and cervical cancer^15^. TCS-monoclonal antibodies have demonstrated specific cytotoxicity to human hepatoma cells *in vitro*^16^, implying the potential application for target treatment of human cancer. TCS also has an anti-virus effect on different human viruses, such as human immunodeficiency virus (HIV), hepatitis B virus (HBV) and herpes simplex virus (HSV)^17^. It was previously reported that TCS could trigger plant defence against different pathogens, such as *Fusarium* *graminearum*^18^, *Pythium* *aphanidermatum*^19^, African cassava mosaic virus (AcMV)^20^, turnip mosaic virus (TuMV)^21^, and tobacco mosaic virus (TMV)^22^. Therefore, *T*. *kirilowii* is an important medical plant and a genetic resource to obtain plant resistance genes.

Plant regeneration *via* somatic embryogenesis (SE) is often adopted in germplasm preservation and establishing high-efficiency transformation systems with advantages including: producing genetically modified plantlets from single cells to avoid mosaics and generally saving time and labour, resulting in high propagation rates and embryogenic cells suitable for continuous suspension culture^23^. Although regeneration systems from *T*. *kirilowii* shoots^24^, root segments, and tips^25^ *via* organogenesis have been previously reported, high-frequency *T*. *kirilowii* regeneration through SE has not been established. In this study, with the optimization of pH values and concentrations of plant growth regulators (PGRs), a high-efficiency regeneration system was established in *T*. *kirilowii*, and rhizoid tubers (RTBs), a novel SE structure, were first observed and named. To our knowledge, this type of SE structure has not previously been reported. It was also found that lower pH values of a medium could significantly promote the induction of rhizoids and RTBs, and further contribute to the high efficiency of SE and regeneration in *T*. *kirilowii*.

## Results

### Rhizoids were first induced from stem, leaf, and root explants in *T*. *kirilowii* by adding NAA to the medium; lower pH values significantly promoted rhizoid induction

**Figure 1:**
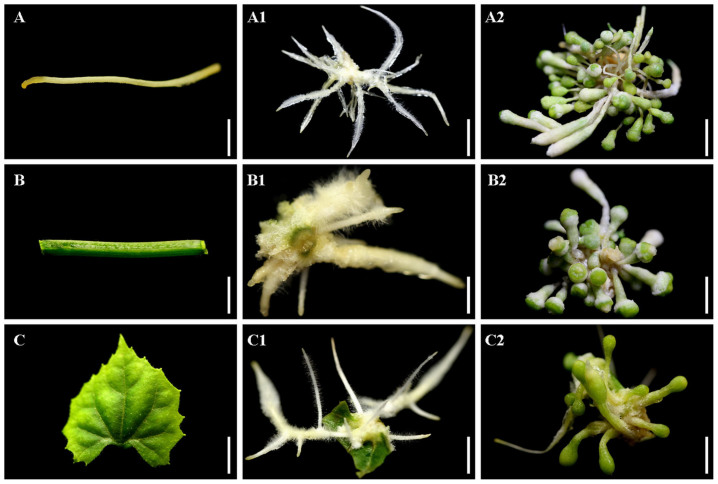
Induction of rhizoids and RTBs from root, stem, and leaf explants of *Trichosanthes kirilowii*. (A) Root explant. (A1) Rhizoids induced from a root explant. (A2) RTBs induced from a root explant. (B) Stem explant. (B1) Rhizoids induced from a stem explant. (B2) RTBs induced from a stem explant. (C) Leaf explant. (C1) Rhizoids induced from a leaf explant. (C2) RTBs induced from a leaf explant. Scale bars for (A, B, and C), 250 μm. Scale bars for (A1, B1, and C1), 1 cm. Scale bars for (A2, B2, and C2), 100 μm.

Two auxin analogues, NAA and 2,4-D, with a concentration series of 0, 0.5, 1.0, and 1.5 mg/L, were used to optimize PGR conditions for the induction of rhizoids. Without NAA and 2,4-D in the medium, no rhizoids were induced (Table 1), suggesting that adding PGR is necessary for rhizoid induction. For all of the 2,4-D supplementary concentrations, no rhizoids were induced, indicating that 2,4-D is not suitable for rhizoid induction in *T*. *kirilowii*. For the NAA supplements, a concentration of 1.0 mg/L resulted in significantly higher average numbers of induced rhizoids per explant from the root, stem, and leaf explants (31.03, 18.90, and 8.03, respectively) (Table 1) than other tested concentrations. For the supplementary NAA concentrations of 0.5, 1.0, and 1.5 mg/L, the rates of rhizoid induction from different explants exhibited the following sequence: root > stem > leaf. With the optimal NAA supplement, three weeks after the inoculation of three types of explants (root, stem, and leaf) (Fig. 1 A, B, and C), small white rhizoids were induced from all three types of explants and developed into a cluster of rhizoids surrounded by many hair-like bodies (Fig. 1 A1, B1, and C1).

With 1.0 mg/L NAA supplement in media, the medium pH values were optimized to check whether they affect the induction of rhizoids. Among pH values of 3.0, 4.0, 5.0, 6.0, 7.0, 8.0, and 9.0, media of pH 4.0 had significantly higher average numbers of induced rhizoids per explant (Table 2). Media of pH 3.0 could not be solidified, and no rhizoids were induced. Root, stem, and leaf explants on media of pH 4.0 had the highest numbers of induced rhizoids per explant (62.33, 40.17, and 11.53, respectively) (Table 2). Except for pH 3.0, which gave no rhizoid induction, other pH values exhibited the following sequence for the average numbers of induced rhizoids per explants: 4.0 > 5.0 > 6.0 > 7.0 > 8.0 > 9.0 (Table 2) for each type of explant. This suggests that, within a suitable range of pH values (pH 4.0–9.0), a lower pH value increases the induction of rhizoids in *T*. *kirilowii*.

### RTBs were induced from rhizoids by adding TDZ to media and lower pH values significantly promoted induction

Rhizoids induced from MS media (pH 5.8) with an optimal supplement of NAA (1.0 mg/L) were transferred to MS media (pH 5.8) containing TDZ to form RTBs. Compared with 10 and 30 mg/L TDZ supplements, the 20 mg/L TDZ supplement resulted in significantly higher RTB induction rates for root, stem, and leaf explants (Table 3), for which all of the rhizoids developed into an RTB. No RTB was induced from rhizoids without adding TDZ (Table 3), suggesting that TDZ is necessary for RTB induction. For 10 and 30 mg/L TDZ supplements, more than 50% of rhizoids failed to develop into RTBs (Table 3). This indicated that 20 mg/L is the best concentration of TDZ for RTB induction. Using the optimized NAA supplement (1.0 mg/L) and pH value (4.0), induced rhizoids from three types of explants were transferred to MS media containing 20 mg/L TDZ but with different pH values (3.0, 4.0, 5.0, 6.0, 7.0, 8.0, and 9.0), and the effects of pH values on RTB induction were analysed. Rhizoids on pH 3.0 media failed to develop into RTBs. On media with pH 4.0, 100% of rhizoids developed into RTBs, with each rhizoid developing into one RTB (Table 4). The trend of the effect of pH values on the induction of RTBs was consistent with that of pH values on rhizoid induction. With increasing pH value (from 4.0 to 9.0), the percentage of rhizoids developing into RTBs decreased in the following sequence: 4.0 > 5.0 > 6.0 > 7.0 > 8.0 > 9.0 (Table 4) for the same type of explant. This suggests that pH 4.0 is the best medium pH value for satisfying both rhizoid and RTB induction.

### Identification and morphological analysis of RTBs

**Figure 2:**
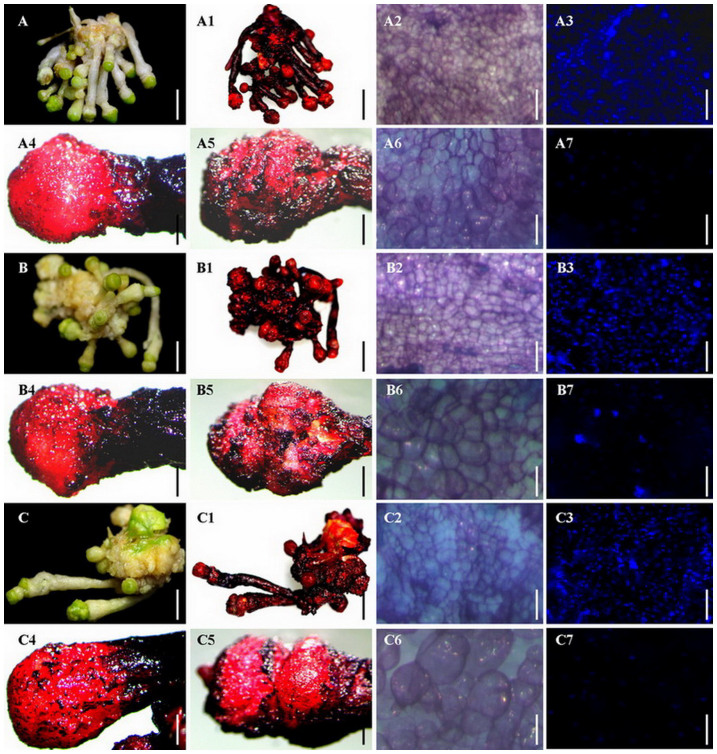
Identification and morphological analysis of RTBs induced from rhizoids of root, stem and leaf explants. (A) Root explant. (A1) RTB identification by staining with acetocarmine and Evans blue. (A2) Morphology and arrangement of RTB embryogenic cells by staining with borax-toluidine blue. (A3) Nuclei of RTB embryogenic cells stained with DAPI and observed in dark-field lighting. (A4) RTB at an early developmental stage. (A5) RTB at a late developmental stage. (A6) Morphology and arrangement of non-embryogenic cells of RTBs stained with borax-toluidine blue. (A7) Nuclei of non-embryogenic cells of RTBs stained with DAPI and observed in dark-field lighting. (B) Stem explant. (B1) RTB identification by staining with acetocarmine and Evans blue. (B2) Morphology and arrangement of RTB embryogenic cells by staining with borax-toluidine blue. (B3) Nuclei of RTB embryogenic cells stained with DAPI and observed in dark-field lighting. (B4) RTB at an early developmental stage. (B5) RTB at a late developmental stage. (B6) Morphology and arrangement of non-embryogenic cells of RTBs by staining with borax-toluidine blue. (B7) Nuclei of non-embryogenic cells of RTBs stained with DAPI and observed in dark-field lighting. (C) Leaf explant. (C1) RTB identification by staining with acetocarmine and Evans blue. (C2) Morphology and arrangement of RTB embryogenic cells by staining with borax-toluidine blue. (C3) Nuclei of RTB embryogenic cells stained with DAPI and observed in dark-field lighting. (C4) RTB morphology at an early developmental stage. (C5) RTB morphology at a late developmental stage. (C6) Morphology and arrangement of non-embryogenic cells of RTBs by staining with borax-toluidine blue. (C7) Nuclei of non-embryogenic cells of RTBs stained with DAPI and observed in dark-field lighting. Scale bars for (A, A1, B, B1, C, and C1), 0.5 cm. Scale bars for (A2, A3, A4, A5, A6, A7, B2, B3, B4, B5, B6, B7, C2, C3, C4, C5, C6, and C7), 500 μm.

Double staining with acetocarmine and Evans blue was employed to analyse whether the induced RTBs were mainly composed of embryogenic tissue. With double staining, embryogenic tissue, in which cells reproduce and metabolize quickly, was stained by both dyes and appeared bright red, while non-embryogenic tissue, in which cells could not be stained with acetocarmine because of their lower metabolism and reproduction, was stained only by Evans blue and appeared blue. This showed that the induced RTBs were in the shape of a ball and were mainly composed of embryogenic tissue in red with a small amount of non-embryogenic tissue in dark blue on the surface (Fig. 2 A1, A4, and A5; B1, B4, and B5; C1, C4, and C5), suggesting that RTB is a kind of special SE structure with the potential to develop into stem embryos. To our knowledge, this is the first observation of RTB; therefore, it is considered as a novel SE structure.

By using microscopic squash technology, together with DAPI staining (Fig. 2 A3 and A7; B3 and B7; C3 and C7) and borax-toluidine blue staining (Fig. 2 A2 and A6; B2 and B6; C2 and C6), embryogenic cells of RTBs were observed to be closely arranged with a thick cytoplasm occupying a large part of the RTB, while non-embryogenic callus cells were much looser in arrangement than embryogenic cells (Fig. 2 A2, B2, and C2). This further confirmed that RTBs are a kind of SE structure.

### Internal structures of RTBs

**Figure 3:**
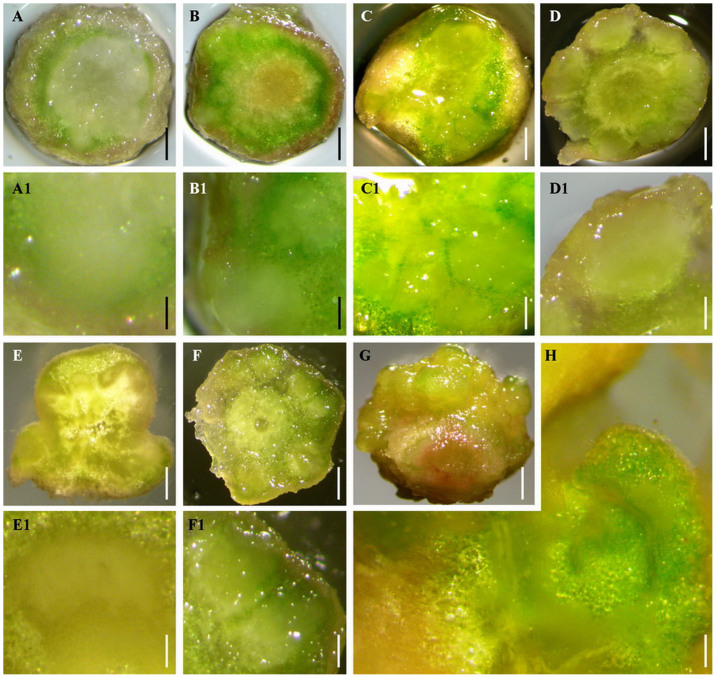
Free-hand slices of RTBs showing the internal structures of RTBs, and an RTB at late developmental stages (G) showing papilla formation at the surface. (A–D and A1–D1) Transverse-section slices of RTBs at different developmental stages. (A) Early stage, embryoids start to form from cortex cells. (B) Early-middle stage, multiple embryoids grow out from cortex. (C) Middle-late stage, embryoids become larger and together with surrounding exodermis form papillae at the surface of RTBs. (D) Late stage, embryoids burst the exodermis and grow out. (A1) Enlarged view of A showing embryoids at an early stage. (B1) Enlarged view of B showing embryoids at an early-middle stage. (C1) Enlarged view of C showing embryoids at middle and late stages. (D1) Enlarged view of D showing embryoids at a late stage. (E, F, H, E1, and F1) Longitudinal-section slices of RTBs at different developmental stages. (E) Early stage. (F) Late stage. (G) RTB at a late stage with papillae formed at the surface of RTB. (H) Spontaneously germinated embryoid at a late stage of RTB development. (E1) Enlarged view of E showing embryoids at an early stage. (F1) Enlarged view of F showing embryoids at a late stage. Scale bars for (A, B, C, D, E, F, and G), 0.3 cm. Scale bars for (A1, B1, C1, D1, E1, and F1), 0.05 cm. Scale bar for H, 0.01 cm.

**Figure 4:**
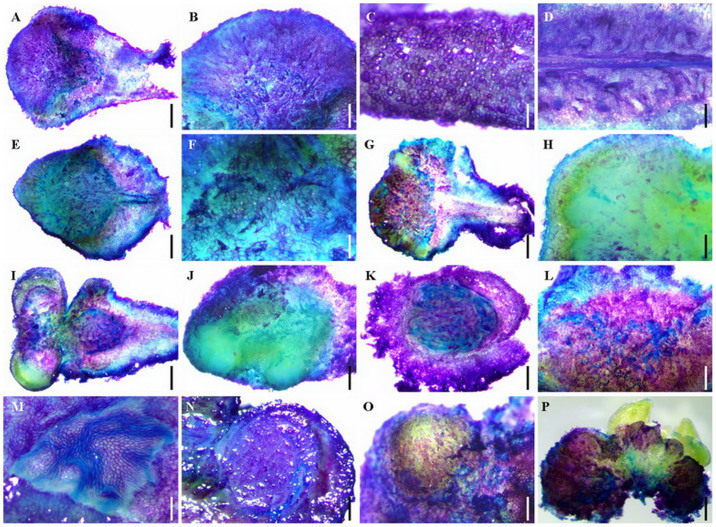
RTBs and their free-hand slices stained with borax-toluidine blue showing the internal structures and macroscopic morphology of RTBs. (A–D) RTBs at an early developmental stage. (A) Longitudinal-section slices of RTBs at an early developmental stage. (B) RTB cap. (C) The cortex cells of rhizoids after peeling the exodermis. (D) The conducting tissue of rhizoids after peeling the partial cortex. (E) RTBs formed at an early developmental stage. (F) Enlarged view of E showing an early embryoid rudiment with rapid cell division. (G) Embryoids at a middle-late developmental stage. (H) Embryoids at a middle-late developmental stage (indigo). (I) Longitudinal-section slices of RTBs at a late developmental stage. (J) Embryoids burst exodermis and grow out. (K) Free-hand slices showing one embryoid at a late developmental stage. (L) Non-embryogenic tissue in RTB was stained purple. (M) Embryoid in RTB at a late developmental stage after peeling the exodermis of the RTB. (N) Embryoid in RTB at a late developmental stage after removing about half of the exodermis. (O) Embryoid covered with exodermis in RTB at a late developmental stage. (P) RTB at a late developmental stage with germinating embryoids. Scale bars for (A, E, G, H, I, J, K, L, N, O, and P), 0.3 cm. Scale bar for B, 0.15 cm. Scale bars for (C, D, F, and M), 100 μm.

In order to analyse the internal structure of RTBs, free-hand slices demonstrating transverse and longitudinal sections of RTBs were directly observed and stained with borax-toluidine blue for further observation of the arrangement of RTB cells. The direct observation of slices without staining showed that embryoids were initially induced from the cortex cells of RTBs (Fig. 3 A, A1, B, and B1), it also demonstrated that multiple embryoids were formed and closely arranged in RTBs at different developmental stages, and developed with the development of RTB (Fig. 3 C, C1, D, D1, E, E1, F, and F1). At the late stage of development, multiple papillae were formed on the surface of RTBs (Fig. 3 G), and free-hand slices of the late-stage RTBs revealed that the papillae were composed of developed and germinated embryoids (Fig. 3 H). The slices were stained with borax-toluidine blue, which caused non-embryogenic cells to be coloured purple and dark blue with clear cell outlines, while the embryogenic cells were indigo. The observation of stained slices revealed the composition of embryogenic and non-embryogenic cells in RTBs (Fig. 4) and confirmed the origin and development of embryoids in RTBs (Fig. 4), further suggesting that RTBs are a kind of SE structure.

### Histological detection revealed the endogenous origin and development of embryoids from rhizoids to RTBs

**Figure 5:**
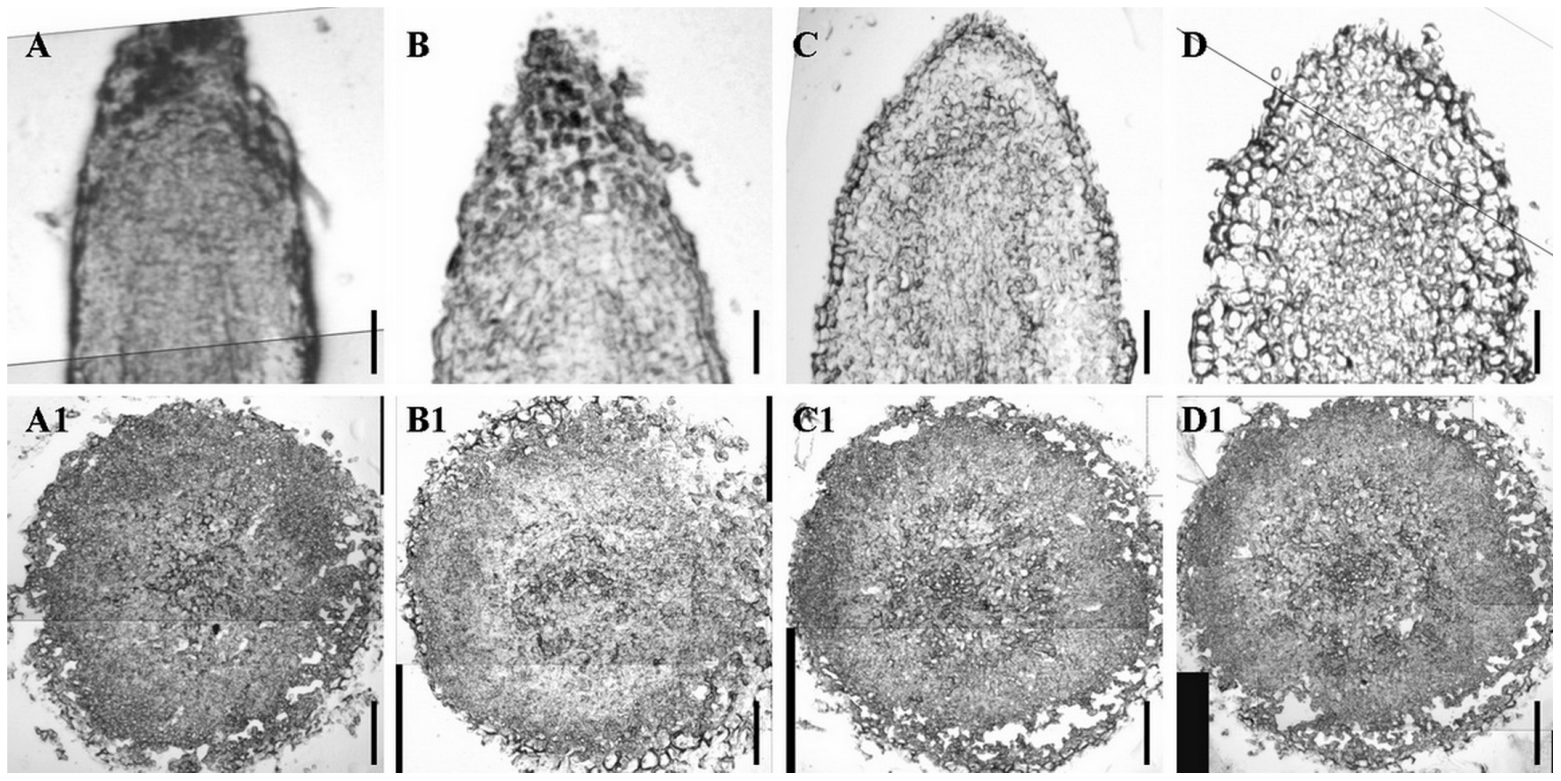
Microscopic images of frozen sections of *Trichosanthes kirilowii* rhizoids at different induction stages. (A) Longitudinal section of a rhizoid. (A1) Transverse section of a rhizoid showing cortex and potential cell division pool at an early stage. (B) Longitudinal section of a rhizoid after one-day incubation on RTB induction medium. (B1) Transverse section of a rhizoid after one-day incubation on RTB induction medium, showing cortex and potential fast-cell-division zone (FCDZ) at an early stage. (C) Longitudinal section of a rhizoid after two-day incubation on RTB induction medium. (C1) Transverse section of a rhizoid after two-day incubation on RTB induction medium, showing cortex and potential FCDZ at an early stage. (D) Longitudinal section of a rhizoid after three-day incubation on RTB induction medium. (D1) Transverse section of a rhizoid after three-day incubation on RTB induction medium, showing cortex and potential FCDZ at an early stage. Scale bars for (A, A1, B, B1, C, C1, D, and D1), 300 μm.

**Figure 6:**
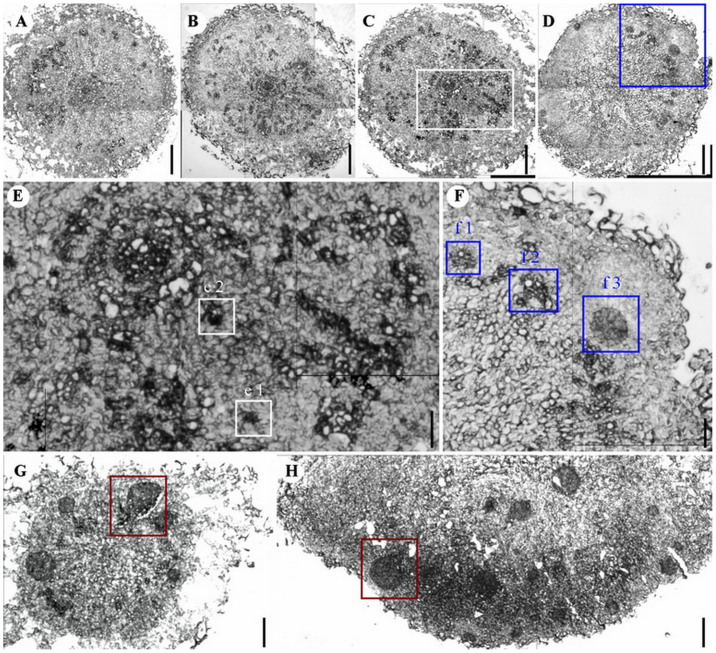
Microscopic images of frozen sections of *Trichosanthes kirilowii* late-stage rhizoids and RTBs at different developmental stages. (A) Transverse section of rhizoid after eight-day incubation on RTB induction medium, showing the cortex and potential fast-cell-division zone (FCDZ). (B) Transverse section of rhizoid after ten-day incubation on RTB induction medium, showing the cortex and potential FCDZ. (C) Transverse section of rhizoid after 12-day incubation on RTB induction medium, showing the cortex and potential FCDZ. (D) Transverse section of rhizoid after 14-day incubation on RTB induction medium, showing the cortex, potential FCDZ, and embryoid rudiment. (E) Enlarged view of C showing proembryos (Boxes e1 and e2). (F) Enlarged view of D showing transitional structures between proembryos and globular embryos (Boxes f1 and f2), and a globular embryo (Box f3). (G) Transverse section of a late-stage RTB with multiple formed embryoids (a heart-torpedo embryo in box). (H) Embryoids (a heart embryo in box) at different developmental stages formed in the RTB cortex. Scale bars for (A, B, C, and D), 300 μm. Scale bars for (E), 100 μm. Scale bars for (F), 80 μm. Scale bars for (H), 30 μm. Scale bars for (G), 300 μm.

The frozen section technique was applied for analysing the development of rhizoids, and the origin and development of RTBs, through longitudinal and transverse sections of the “cap” of rhizoids and RTBs. The longitudinal and transverse sections of rhizoids suggest that the internal structures of RTBs, especially the inside cortex cells (Fig. 5 A and A1), experienced obvious changes two days (Fig. 5 B and B1), four days (Fig. 5 C and C1), and six days (Fig. 5 D and D1) after rhizoids were transferred to the RTB induction medium and cultivated under high light. The darker coloured, vigorous cell division zones were distributed in the thickened cortexes (Figs. 5 D1 and 6 A–D). RTB proembryos started to appear 12 days after rhizoids were transferred to RTB induction medium (Fig. 6 C and E). RTB embryoids started to be formed 14 days after rhizoids were transferred to RTB induction medium (Fig. 6 D and F) and the formed globular, heart, and heart-torpedo embryos grew along the marginal zone of the cortex (Fig. 6 D, F, G, and H). This indicated that RTB embryoids were derived from the cortex cells of rhizoids and sequentially induced, exhibiting different sizes (Fig. 6 A, B, C, and D). It also demonstrated that one individual RTB could contain multiple embryoids at different developmental stages (Fig. 6 G and H).

### Plantlets could develop from *in vitro* and *in vivo* RTBs

**Figure 7:**
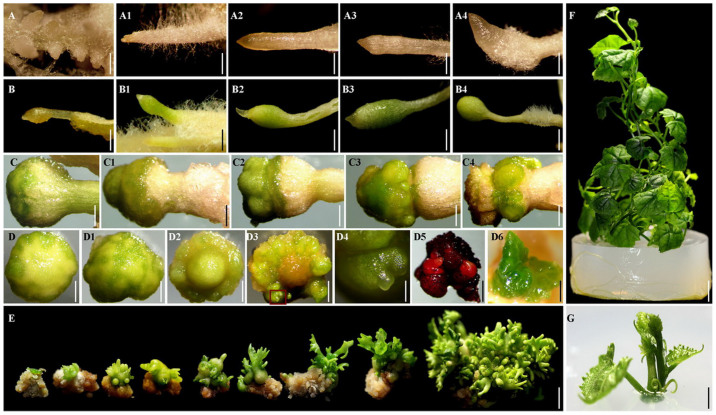
The regeneration process of *Trichosanthes kirilowii* *via* somatic embryogenesis (SE) involving RTBs, a new kind of SE structure. (A–A4) Rhizoids induced on root explants. (A) Rhizoid primordia induced on a root explant 2 days post inoculation (dpi). (A1) Rhizoids induced on a root explant 3 dpi. (A2) One induced rhizoid on a root explant 5 dpi. (A3) One induced rhizoid on a root explant 7 dpi. (A4) One induced rhizoid on a root explant 9 dpi. (B–B4) Induced RTBs at an early developmental stage. (B) One induced RTB 1 day post transferring rhizoids to RTB induction medium (dpt). (B1) One induced RTB 3 dpt. (B2) One induced RTB 10 dpt. (B3) One induced RTB 12 dpt. (B4) One induced RTB 14 dpt. (C–C1) Induced RTBs at an early-middle developmental stage. (C) One induced RTB 25 dpt. (C1) One induced RTB 30 dpt. (C2–C4) Induced RTBs at a middle-late developmental stage. (C2) One induced RTB 35 dpt. (C3) One induced RTB 40 dpt. (C4) One induced RTB 45 dpt. (D–D2) Polar views of RTBs at an early-middle developmental stage, D (25-day induction), D1 (28-day induction), and D2 (30-day induction). (D3) Polar views of RTBs at a late developmental stage (45-day induction). (D4) Enlarged view of D3 showing formed embryoids. (D5) The formed embryoids stained red by acetocarmine and Evans blue. (D6) Macroscopic morphology of formed embryoid. (E) The formation process of multiple plantlets from an *in vitro* RTB. (F) A regenerated seedling. (G) A regenerated plant. Scale bars for (A, A1, A2, A3, A4, B, B1, B2, B3, and B4), 1 mm. Scale bars for (C, C1, C2, C3, C4, D, D1, D2, D3, D4, and D5), 1.5 mm. Scale bar for D6, 5 mm. Scale bars for (E, F, and G), 0.5 cm.

Two methods were used to induce plantlets from *in vitro* and *in vivo* RTBs in *T*. *kirilowii*. *In vivo* RTBs on the same induction media as that for inducing RTBs could spontaneously develop into multiple plantlets at a lower frequency, approximately 37.77%, 33.47%, and 31.07% for RTBs from root, stem, and leaf explants, respectively. In order to increase the frequency of plantlet induction, *in vitro* RTBs separated from explants were transferred to MS media supplemented with BAP (Table 5). The result of BAP optimization indicated that *in vitro* RTBs on MS medium supplemented with 5.0 mg/L BAP developed into multiple plantlets (Fig. 7) with significantly higher frequencies for root, stem, and leaf explants (97.57%, 95.90%, and 95.97%, respectively) than those on media with other concentrations of BAP (Table 5). The result that one individual RTB could develop into multiple plantlets is different from the usual scenario of SE, in which one SE structure often individually develops and forms one plantlet^26^,^27^.

## Discussion

The optimal supplement of PGRs is important for the success of SE and regeneration in plants^23^,^28^,^29^. In the present study, SE in *T*. *kirilowii* was achieved through two steps supplemented with NAA and TDZ, respectively. For the first step, rhizoids were induced by adding NAA at an optimal concentration in media, and for the following step RTBs were induced from the rhizoids by adding TDZ at an optimal concentration in media. Auxin analogues of NAA and 2,4-D were tested for rhizoid induction, and 2,4-D did not induce rhizoids in *T*. *kirilowii*, whereas NAA resulted in successful rhizoid induction. This suggests that different auxin analogues have different effects on rhizoids in *T*. *kirilowii*. Therefore, the right selection and concentration optimization of PGRs is a prerequisite for successful SE. Light conditions also played an important role in the two steps of SE in *T*. *kirilowii*; the explants were kept in the dark for rhizoid induction, whereas high light benefited the induction of RTBs. High light conditions are different from those of previous reports in which moderate light was employed for inducing somatic embryos^27^,^30^. In this paper, we found that a lower pH value (4.0) surprisingly resulted in significantly higher rates for the inductions of rhizoids and RTBs than the normally adopted pH value (5.8), and, for the pH value series of 4.0, 5.0, 6.0, 7.0, 8.0, and 9.0, increasing pH decreased the induction rates of rhizoids and RTBs. However, the inductions of rhizoids and RTBs failed on media with pH 3.0; it seems that this is too low to induce rhizoids and RTBs. We also found that media with pH 3.0 could not be solidified, which could be one reason for the failure to induce rhizoids and RTBs on media with pH 3.0. This suggests that a lower pH value benefits rhizoid and RTB induction in *T*. *kirilowii* within a suitable pH range. The mechanism causing these benefits in *T*. *kirilowii* and whether it is universal in other plants need further study.

For rhizoids and RTBs originated from root, stem, and leaf explants of *T*. *kirilowii*, induction frequencies were significantly different in the sequence: root > stem > leaf. This suggests that different organs have different capacities for rhizoid and RTB induction, and root explants are a good choice for SE *via* rhizoid and RTB induction in *T*. *kirilowii*. The capacity ranking among organs of SE *via* RTB in *T*. *kirilowii* is different from that *via* frog egg-like bodies in *Solanum nigrum* (leaf > root > stem)^23^, suggesting that the organ resulting in the highest induction frequency of SE could be different in different SE induction pathways and different plant species.

The observed RTBs are considered to be a novel kind of SE structure for the following reasons: 1) to our knowledge, a tuber-shaped structure (RTB) during SE has not previously been reported; 2) evidence showed that RTBs are composed of embryogenic cells that could developed into embryos; 3) rhizoids first formed in SE mediated by RTB, different from the callus formation in a typical SE; 4) multiple embryos can be formed within an individual RTB, whereas embryos are often individually formed on the callus surface in a typical SE pathway; and 5) RTBs can spontaneously develop into multiple plantlets at a higher frequency than embryos in a typical SE pathway. In addition, we found that mature RTBs had a hard, green outside layer. The layer benefits embryo formation and protects embryos inside, which may play a similar role to the protection layer of artificial seeds. Therefore, RTB could be a good candidate to produce artificial seeds.

*T*. *kirilowii* is a medicinal plant with therapeutic properties belonging to the Cucurbitaceae family, some accessions of which exhibit a high resistance to many phytopathogens, viruses, and insect attacks^20^,^21^,^22^. Therefore, *T*. *kirilowii* can be used not only as medicinal material, but also serve as a resource for disease resistance genes for improving important Cucurbitaceae crops such as cucumber, bitter gourd, watermelon, and pumpkin. The regeneration system *via* SE by RTBs will promote the establishment of transformation systems in *T*. *kirilowii*, which will help increase the content of TCS and other valuable metabolites. The developed system can be used to obtain pathogen-free *T*. *kirilowii* plants because the vascular tissue of somatic embryos is independent from that of the parental explants. Plant regeneration *via* SE by RTBs in *T*. *kirilowii* could give new insight for establishing similar systems in other plants, especially in recalcitrant plants for SE induction and regeneration. However, further studies are needed to ascertain whether RTBs are universal in other plant species.

## Methods

### Plant materials and explant preparation

*T*. *kirilowii* stems with axillary buds were treated with 75% (v/v) ethanol for 30 s, rinsed three times with sterilized distilled water, soaked in 0.1% (v/v) mercury bichloride for 8–10 min, and rinsed five times with sterilized distilled water. The sterilized stem segments were inserted in Murashige and Skoog (MS) medium^31^ supplemented with 0.1 mg/L gibberellic acid (GA_3_), 30 mg/L sucrose, and 7.8 g/L agar (pH 5.8) to obtain axillary shoots.

When the axillary shoots were approximately 1–2 cm long, they were separated and transplanted onto MS medium and cultivated at 25°C with a 16 h photoperiod (180 μmol·m^−2^·s^−1^). After 2–3 weeks of cultivation, the shoots developed into plantlets with roots. Then, the leaves or leaf discs of about 1 cm^2^ in area and cut root and stem segments of about 1 cm length (for stem segments, axillary buds were avoided) were excised from the plantlets as explants for the induction of rhizoids.

### Induction of rhizoids and RTBs

For optimizing supplementary PGRs for rhizoid induction, leaf, root, and stem explants were placed on MS media with 30 g/L sucrose and 7.8 g/L agar, pH 5.8, supplemented with 2,4-dichlorophenoxyacetic acid (2,4-D) and 1-naphthaleneacetic acid (NAA) with the concentration series 0, 0.5, 1.0, and 1.5 mg/L. The explants were cultivated at 25 ± 1°C in the dark to induce satisfactory rhizoids. The effects of pH values (3.0, 4.0, 5.0, 6.0, 7.0, 8.0, and 9.0) on rhizoid induction were investigated with the optimal concentration of PGRs. For the induction of RTBs, the rhizoid clusters induced on media containing 1.0 mg/L NAA were transplanted onto MS medium (pH 5.8) supplemented with N-phenyl-N′-1, 2, 3-thiadiazol-5-ylurea (thidiazuron, TDZ) (at 0, 10.0, 20.0, and 30.0 mg/L) and cultivated under light conditions (180 μmol·m^−2^·s^−1^). The effects of pH values (3.0, 4.0, 5.0, 6.0, 7.0, 8.0, and 9.0) on RTB induction were also analysed. The rhizoids and RTBs at different developmental stages were recorded using a digital camera (EOS 600D, Canon Inc., Japan) and a stereomicroscope (SMZ800, Nikon Corporation, Japan).

### Histochemical and histological analyses of rhizoids and RTBs

To confirm the presence of embryonic cells in RTBs, double staining with acetocarmine and Evans blue^23^,^32^ was used to distinguish embryonic tissue from calluses using images taken with a digital camera (EOS 600D, Canon Inc., Japan) in which embryogenic cells were stained bright red and non-embryogenic calluses were stained dark blue.

Staining with 4′,6-diamidino-2-phenylindole (DAPI) was used to detect the nuclei of embryonic and callus cells, following a previously published method^23^,^32^. A thin slice of RTB tissue was placed on a slide and photographed with dark-field illumination using a digital fluorescence microscope (BX 61, Olympus Corporation, Japan). Cell outlines of rhizoids and RTBs were observed using borax-toluidine blue staining, according to our published protocol^32^, and images were taken using an optical microscope (BX 41, Olympus Corporation, Japan).

The microscopic frozen sections of rhizoids and RTBs at different developmental stages were created following a previously published method^23^, and imaged using an optical microscope (BX 41, Olympus Corporation, Japan).

### Analyses of the internal structures of RTBs with single staining of free-hand slices

The free-hand slices (about 1 mm in thickness) cut from RTBs were put in the water and imaged using a stereomicroscope (SMZ800, Nikon Corporation, Japan). For single staining, the cut slices were immersed in staining solution (1% borax-toluidine blue dissolved in 1% sodium tetraborate as solvent) for 5 s, and rinsed with distilled water to remove residue dye. Then, the stained slices were imaged under light-field conditions using a stereomicroscope (SMZ800, Nikon Corporation, Japan).

### Plantlet formation from RTBs

Two approaches were employed to form plantlets from RTBs. In the first approach, *in vivo* RTBs were kept on the same MS medium (supplemented with 20 mg/L TDZ) as that for the induction of RTBs to spontaneously form plantlets. In the second approach, *in vitro* RTBs were placed on MS medium (pH 5.8) supplemented with 5.0 mg/L 6-benzylaminopurine (BAP). For plantlet formation, both *in vitro* and *in vivo* RTBs were cultivated at 25°C under a 16-h photoperiod with a light intensity of 180 μmol·m^−2^·s^−1^ and subcultured monthly. When plantlets grew to 1–2 cm, long they were separated and transferred to the rooting medium for root formation. To evaluate the frequency of regenerated plantlets from RTBs, 30 replicates of every 10 RTBs were set.

### DAPI staining and microscopic observation

Based on a previously published approach^32^, DAPI staining was conducted to visualize cell nuclei in images taken using a digital fluorescence microscope (BX 61, Olympus Corporation, Japan) with a mirror unit (U-MNU2), a dichroic mirror (DM400), an excitation filter (BP360), and a barrier filter (BA420).

### Statistical analysis

The digital data was analysed using analysis of variance (ANOVA) using SPSS 10.0, with 99% and 95% confidence intervals.

## Author Contributions

K.D.X. and C.W.L. conceived and designed the experiments. K.D.X., Y.X.C., J.Z., P.L.W., J.X.W., Y.Y.L., X.W.W. and W.W. performed the experiments. K.D.X., G.X.T. and C.W.L analyzed the data. K.L., Y.Z., D.S.Y., L.B.L., Y.L. and S.Y.M. contributed reagents/materials/analysis tools. K.D.X. and C.W.L. wrote the paper.

## Figures and Tables

**Table 1:** Effect of NAA on induction of rhizoids from *Trichosanthes kirilowii* root, stem, and leaf explants

NAA (mg/L)	Average numbers of induced rhizoids per explant		
	Root	Stem	Leaf
0	0.00 ± 0.00 jH	0.00 ± 0.00 jH	0.00 ± 0.00 jH
0.5	10.10 ± 0.27 cC	5.07 ± 0.14 fE	3.77 ± 0.27 hF
1.0	31.03 ± 0.28 aA	18.90 ± 0.24 bB	8.03 ± 0.13 eD
1.5	7.47 ± 0.18 dD	4.37 ± 0.18 gF	2.43 ± 0.18 iG

Note: The mean and standard error per treatment were calculated from 300 explants from 30 Petri dishes (as 30 replicates). Capital and lowercase letters indicate a significant difference at the 1% and 5% probability levels, respectively. Significant differences were analysed with Duncan's test using SPSS 10.0.

**Table 2:** Effect of pH values on the induction of rhizoids from *Trichosanthes kirilowii* root, stem, and leaf explants supplemented with 1 mg/L NAA in media

pH values	Average numbers of induced rhizoids per explant		
	Root	Stem	Leaf
3.0	0.00 ± 0.00 pN	0.00 ± 0.00 pN	0.00 ± 0.00 pN
4.0	62.33 ± 0.67 aA	40.17 ± 0.32 bB	11.53 ± 0.27 iH
5.0	38.80 ± 0.43 cC	29.73 ±0.54 eE	8.17 ± 0.14 kJ
6.0	31.03 ± 0.28 dD	18.50 ± 0.27 fF	8.07 ± 0.12 kJ
7.0	17.57 ± 0.22 gF	10.33 ±0.36 jI	4.53 ± 0.21 mK
8.0	13.43 ± 0.29 hG	5.53 ± 0.20 lK	2.23 ± 0.17 nL
9.0	1.90 ± 0.25 nLM	0.97 ± 0.16 oMN	0.43 ± 0.16 opN

Note: The mean and standard error per treatment were calculated from 300 explants from 30 Petri dishes (as 30 replicates). Capital and lowercase letters indicate a significant difference at the 1% and 5% probability levels, respectively. Significant differences were analysed with Duncan's test using SPSS 10.0.

**Table 3:** Effect of TDZ on RTB induction from rhizoids of *Trichosanthes kirilowii* root, stem, and leaf explants

TDZ (mg/L)	Average numbers of induced RTBs per explant		
	Root	Stem	Leaf
0	0.00 ± 0.00 hH	0.00 ± 0.00 hH	0.00 ± 0.00 hH
10.0	10.60 ± 0.32 cC	5.37 ± 0.19 eE	4.57 ± 0.24 fF
20.0	30.73 ± 0.30 aA	19.07 ± 0.25 bB	8.13 ± 0.18 dD
30.0	8.07 ± 0.20 dD	4.57 ± 0.18 fF	2.87 ± 0.25 gG

Note: The mean and standard error per treatment were calculated from 300 explants from 30 Petri dishes (as 30 replicates). Capital and lowercase letters indicate a significant difference at the 1% and 5% probability levels, respectively. Significant differences were analysed with Duncan's test using SPSS 10.0.

**Table 4:** Effect of pH values on RTB induction from rhizoids of *Trichosanthes kirilowii* root, stem, and leaf explants supplemented with 20 mg/L TDZ in media

pH values	Average numbers of induced RTBs per explant		
	Root	Stem	Leaf
3.0	0.00 ± 0.00 oM	0.00 ± 0.00 oM	0.00 ± 0.00 oM
4.0	61.50 ± 0.68 aA	40.70 ± 0.43 bB	11.43 ± 0.34 iG
5.0	38.80 ± 0.55 cC	29.77 ± 0.55 eD	8.17 ± 0.17 jH
6.0	30.73 ± 0.35 dD	18.50 ± 0.43 fE	7.60 ± 0.22 jH
7.0	17.40 ± 0.25 gE	11.10 ± 0.42 iG	4.77 ± 0.27 lJ
8.0	13.40 ± 0.37 hF	6.07 ± 0.27 kI	2.53 ± 0.19 mK
9.0	2.00 ± 0.25 mKL	1.00 ± 0.15 nLM	2.13 ± 0.28 mKL

Note: The mean and standard error per treatment were calculated from 300 explants from 30 Petri dishes (as 30 replicates). Capital and lowercase letters indicate a significant difference at the 1% and 5% probability levels, respectively. Significant differences were analysed with Duncan's test using SPSS 10.0.

**Table 5:** Frequencies of plantlet induction from *in vivo* and *in vitro* RTBs of *Trichosanthes kirilowii* root, stem, and leaf explants

*In vitro* RTBs on media with BAP (mg/L)	*In vivo* RTBs on media with TDZ (mg/L)	Frequency of plantlet induction (%)		
		Root	Stem	Leaf
	20	37.77 ± 0.57 gF	33.47 ± 0.36 hG	31.07 ± 0.33 iH
0		37.13 ± 0.88 gF	33.43 ± 0.37 hG	31.97 ± 0.29 hiGH
2.5		75.13 ± 1.33 bB	70.47 ± 0.37 dC	72.13 ± 0.32 cC
5.0		97.57 ± 0.34 aA	95.90 ± 0.45 aA	95.97 ± 0.44 aA
7.5		67.67 ± 0.76 eD	64.80 ± 0.67 fE	63.30 ± 0.37 fE

Note: Frequency of plantlets induced from RTBs refers to the proportion of RTBs forming plantlets out of the total number of investigated RTBs. The mean and standard error per treatment were calculated from 300 explants from 30 Petri dishes (as 30 replicates). Capital and lowercase letters indicate a significant difference at the 1% and 5% probability levels, respectively. Significant differences were analysed with Duncan's test using SPSS 10.0.
